# Modeling the variables that influence substance consumption of people who experience homelessness in Colombia

**DOI:** 10.3389/fsoc.2025.1474113

**Published:** 2025-07-30

**Authors:** Leandro González Támara, Sandra Patricia Barragán Moreno

**Affiliations:** ^1^Superior School of Public Administration, Territorial Cundinamarca, Fusagasugá, Colombia; ^2^Academic Area of Basic Sciences and Modeling, Faculty of Natural Sciences and Engineering, University of Bogotá Jorge Tadeo Lozano, Bogotá, Colombia

**Keywords:** statistical model, homeless person, psychotropic drugs, drug abuse, population census

## Abstract

**Background:**

Homelessness in Colombia is a critical social issue that is strongly associated with psychoactive substance use. This study aims to model the variables influencing substance use among individuals experiencing homelessness in Colombia, offering insights to inform public policy design.

**Methods:**

This research draws on data from the National Administrative Department of Statistics (DANE) censuses conducted in 2017, 2019, and 2021. A two-stage quantitative methodology was applied: (1) descriptive analysis of the demographic and socioeconomic characteristics of the homeless population, and (2) predictive modeling using random forest algorithms to identify key variables associated with substance use. While results reveal strong associations, they do not imply causality. The study focuses on available variables, acknowledging the absence of psychosocial factors and the need for complementary qualitative research.

**Results:**

The analysis identified age and the duration of homelessness as the most influential variables associated with substance use. Descriptive findings revealed that 66–68% of the homeless population reported consuming at least one psychoactive substance, with higher consumption rates observed among younger individuals and those with shorter durations of homelessness. The random forest model demonstrated high predictive accuracy and confirmed the centrality of these variables. Moreover, men were more likely to use substances than women, and both family conflict and prior substance use were key factors contributing to the onset and persistence of homelessness.

**Discussion:**

The findings indicate that substance use is prevalent among homeless individuals in Colombia and shaped by distinct demographic factors. The bidirectional relationship between homelessness and substance use reveals a complex dynamic in which each condition reinforces the other. These results highlight the importance of targeted interventions directed at younger individuals and those recently experiencing homelessness. Future research using system dynamics modeling is recommended to further explore the feedback mechanisms underlying this relationship.

**Conclusion:**

This study offers a detailed analysis of the variables influencing substance use among homeless individuals in Colombia. By identifying age and homelessness duration as critical factors, the research contributes actionable knowledge for the development of evidence-based public policies. Implementing targeted interventions based on these findings may improve the health outcomes and social reintegration of this vulnerable population, ultimately enhancing public health and safety.

## 1 Introduction

Housing accessibility challenges is a social problem suffered by more and more people, and it also represents an invisible and chronic reality that prevents access to a fundamental right. Homelessness involves the failure to comply with many other human rights, such as the right to health, employment, equal treatment or security. This violation of rights is rooted in external poverty, is the crudest expression of inequality, and is clearly linked with the SDGs (Botija et al., 2024, p.2) (own translation).

In Colombia, Law 1,641 of 2013 defines homelessness (or street dwelling) as the condition of individuals who live permanently or temporarily on the streets, irrespective of gender, race, or age (Congreso de la República de Colombia, 2013). Homelessness in Colombia is a complex and multifaceted phenomenon shaped by intersecting social, psychological, and structural drivers. Magwood et al. (2020) characterize it as the absence of stable housing, including living on the streets, in temporary accommodations, or in emergency shelter conditions often driven by economic constraints (Table 1).

**Table 1:** Comparative demographic statistics between heavy and general users.

Variable	General users	Heavy users
Men	17,370 (89.4%)	14,796 (89.4%)
Women	2,060 (10.6%)	1,761 (10.6%)
Sex total	19,430 (54%)	16,557 (46%)
Age	39.2 (13.3)	37.6 (12.2)
Main reason		
Sexual abuse	74 (0%)	65 (0%)
Threat or risk to life or physical integrity	294 (2%)	241 (1%)
Family conflicts or difficulties	5,864 (30%)	4,964 (30%)
Consumption of psychoactive substances.	7,643 (39%)	7,109 (43%)
Economic difficulties	1,439 (7%)	889 (5%)
Lack of work	780 (4%)	466 (3%)
Influence of other people	522 (3%)	469 (3%)
Other	720 (4%)	546 (3%)
For personal taste	1,626 (8%)	1,425 (9%)
Has always lived on the street	108 (1%)	91 (1%)
Victim of armed conflict or displaced	359 (2%)	291 (%)
2017	6,280 (65.8%)	5,365 (56.2%)
2019	8,887 (67.1%)	7,695 (58.1%)
2021	4,263 (68.2%)	3,497 (56.0%)
Total 2017, 2019, 2021	19,430 (66.9%)	16,557 (57.2%)

*Mean (Standard deviation). The percentages do not sum to 100 because each value represents the proportion of homeless individuals classified as either general or heavy consumers within their respective categories. Source: elaborated by the authors.

A significant body of literature (Aldridge, 2020; Doran et al., 2018; Aldridge et al., 2018; Laporte et al., 2018; Barnett and Owusu, 2017) has identified homelessness as being linked to extreme poverty, health risks, violence, lack of essential services, and heightened vulnerability to substance use and mental illness. Cobb (2020) emphasized the profound fragility of people experiencing homelessness, noting that adverse circumstances are magnified by their unstable living conditions. He further cautioned against stereotyping, as views perpetuate marginalization. Similarly, Wagstaff et al. (2023) stressed that stigma and discrimination are among the most significant obstacles homeless individuals face in reintegrating into society (Table 2).

**Table 2:** Main reasons why people began experiencing homelessness and continue living in it.

Reason	Main reason why people began to experience homelessness	Main reason why people persist in the situation of homelessness
Family conflicts or difficulties	6,725 (30.1%)	2,740 (12.3%)
Consumption of psychoactive substances	8,195 (36.6%)	8,216 (36.7%)
Economic difficulties	2,054 (9.2%)	3,149 (14.1%)
Lack of work	1,051 (4.7%)	2,243 (10.0%)
Other	948 (4.2%)	906 (4.1%)
Personal choice	1,807 (8.1%)	2,731 (12.2%)
Has always lived on the street	138 (0.6%)	200 (0.9%)
Total	22,374	22,357

Source: elaborated by the authors.

The vulnerability of this population is further compounded by the high prevalence of psychoactive substance use, a pressing public health concern that can act both as a cause and a consequence of homelessness (Johnson and Chamberlain, 2008). According to Colombia’s Ministerio de Salud y Protección Social (2016, p. 1), a psychoactive substance is:

Any substance introduced into the body by any route of administration (ingested, smoked, inhaled, injected, among others) that produces an alteration in the functioning of the individual’s central nervous system, which modifies consciousness, mood, or thought processes. (Own translation).

Addiction to such substances significantly deteriorates both physical and mental health, exacerbates social marginalization, and complicates the prospects for reintegration (Romero-Plana, 2023; Aldridge et al., 2018).

Homelessness constitutes an extreme form of social exclusion, characterized by marginalization and adverse health outcomes due to poverty and comorbid conditions (Aldridge et al., 2018). This definition includes individuals living in shelters or directly on the streets, as well as those who have experienced unstable housing in the previous 12 months. Studies show that individuals experiencing homelessness display higher levels of substance use compared to the general population (Doran et al., 2018). This also includes those in transitional housing (Barnett and Owusu, 2017) or those facing severe housing instability (Ballard et al., 2023). A broader perspective includes anyone lacking stable, permanent, and adequate housing, or the means to secure such conditions (Magwood et al., 2020).

Understanding the key factors influencing substance use within this population is critical to formulating effective public policies and rehabilitation programs. In Colombia, homelessness stems from multiple causes, such as economic hardship, lack of access to stable housing, and social exclusion (Camargo et al., 2020), which are further exacerbated by mental health disorders and substance dependence (Grigsby et al., 2023; DANE, 2017a, 2019, 2021).

Developing adequate interventions requires a deeper understanding of the lived experiences of this population (Fitzpatrick et al., 2013). Calderón et al. (2020) emphasized the importance of identifying their characteristics to ensure that their rights and needs are adequately addressed (Johnson and Chamberlain, 2008). Limited access to services tailored to their specific needs further increases their vulnerability. For instance, Cawley et al. (2022) investigated the leading causes of death among homeless individuals in San Francisco during the COVID-19 pandemic, revealing that acute toxicity from medications was the most common cause, rather than the virus itself.

Addressing homelessness demands a comprehensive, multi-sectoral approach. While progress has been made, greater efforts are required to implement public policies that take a preventative, person-centered approach across social, educational, economic, labor, and healthcare domains (Agha et al., 2024; Adams et al., 2023). Medellín Solidaria, a strategy led by the Mayor’s Office of Medellín, exemplifies such an effort. It aimed to reduce extreme poverty by promoting social inclusion and providing access to temporary housing, detoxification, and rehabilitation services (Alcaldía de Medellín, 2017).

Health is a critical factor in understanding homelessness. Liu et al. (2022) emphasized that individuals experiencing homelessness face significant barriers to care and suboptimal health service transitions, a view supported by Ballard et al. (2023) and Agha et al. (2024). In Colombia, Camargo et al. (2020) highlighted how extreme poverty, social exclusion, and limited institutional response create structural conditions that reinforce both homelessness and addiction. Substance use is a pervasive and pressing concern, contributing significantly to morbidity and mortality (Coombs et al., 2024; Adams et al., 2023). This cycle perpetuates poverty and exclusion (Aldridge et al., 2018; Romero-Plana, 2023; Laporte et al., 2018).

From the lens of the “right to the city” (Lefebvre, 1968), homeless individuals face not only material deprivation but also structural exclusion from urban citizenship. Roy et al. (2022) demonstrated how social exclusion shapes the daily lives of homeless individuals in urban contexts like Siliguri, India, illustrating the ways in which access to rights is systematically restricted.

Understanding the factors influencing substance use among people experiencing homelessness is essential—not only because it constitutes a major public health issue, but because it impacts a population that is already extremely vulnerable (Wagstaff et al., 2023; Fitzpatrick et al., 2013). Fitzpatrick et al. (2013) propose a critical realist framework, identifying homelessness, substance misuse, and mental health challenges as interrelated outcomes of poverty and early trauma. These processes do not occur in isolation but are embedded within structural dynamics of exclusion, reinforcing vulnerability over time.

Based on these considerations, the guiding research question for this study is: What are the key social, psychological, and structural factors influencing psychoactive substance use among people experiencing homelessness in Colombia, and how do these factors interact? Consequently, the objective is to model the variables that influence psychoactive substance use among this population. The findings aim to inform public policy and support more effective interventions (Alcaldía de Medellín, 2017; Calderón et al., 2020). The modeling approach may yield benefits not only for affected individuals but also for public health and safety systems (Johnson and Chamberlain, 2008; Jaén and Dyner, 2007).

This exploratory study focuses on identifying and modeling the key factors that influence psychoactive substance use among people experiencing homelessness in Colombia. It does not seek to test specific hypotheses about causal relationships but aims instead to inform future research and public policy design.

The article is structured as follows: following this introduction, the theoretical framework defines the conceptual basis of homelessness, presents findings from two bibliometric mappings, and introduces the methodology. The methodology consists of two stages: (1) a description of the homeless population in Colombia based on census data from DANE (2017a, 2019, 2021), and (2) modeling using the random forest algorithm. Results are then presented and discussed, followed by concluding remarks.

## 2 Theoretical framework

To construct the theoretical foundation for this study, this section is divided into two components aligned with the research question and objectives: (1) the conceptual definition of homelessness, recognizing that definitions may vary according to national economic contexts; and (2) the evolution of scientific research on the variables influencing psychoactive substance use among people experiencing homelessness in Colombia.

### 2.1 Conceptualization of homelessness and psychoactive substances

In Spanish, multiple expressions are used to describe people who experience homelessness. These terms have evolved over time, reflecting increased empathy and recognition of homeless individuals as rights-holders. Commonly used terms include *indigente* (indigent) and *persona en situación de calle* (street dweller) in Latin America (DANE, 2017a, 2017b, 2019, 2021). In Spain, terms such as *personas sin hogar* (homeless people) or *en situación de sinhogarismo* (people in a homelessness situation) are prevalent (Botija et al., 2024). In English-speaking countries, common expressions include “homeless individuals” and “street dwellers” (Cobb, 2020; Laporte et al., 2018).

The conceptual definitions of both psychoactive substances and homelessness (as discussed in the introduction) provide a foundation for modeling the bidirectional relationship between homelessness and substance use (DANE, 2021; Aldridge, 2020; Calderón et al., 2020; Barnett and Owusu, 2017; Johnson and Chamberlain, 2008).

This study adopts the conceptual framework developed by Johnson and Chamberlain (2008), which identifies two primary explanatory models: social selection and social adaptation. The social selection model posits that substance use can lead to the loss of employment, social networks, and housing, thereby acting as a trigger for homelessness. Conversely, the social adaptation model argues that the adverse conditions of homelessness—such as insecurity, lack of privacy, limited access to healthcare, and exposure to street cultures where substance use is normalized—can lead individuals to begin or escalate substance use as a coping mechanism.

These two models underscore the bidirectional and reinforcing nature of the relationship between homelessness and substance use. Factors such as the age of onset and duration of homelessness serve as modulators of this relationship. For example, individuals who become homeless during adolescence are more likely to engage in persistent substance use, and prolonged homelessness increases the risk of severe addiction. This framework informs the selection of variables in the statistical model and guides the interpretation of findings.

### 2.2 Evolution of research on variables affecting substance use among the homeless

A scientific mapping was conducted to analyze the evolution of research on variables associated with psychoactive substance use among homeless populations, both globally and within Colombia (Chambergo et al., 2021; Grant and Booth, 2009). This involved a co-occurrence analysis of keywords using documents retrieved from the interdisciplinary SCOPUS database, visualized through network graphs generated with VOSviewer (Donthu et al., 2021). According to van Eck and Waltman (2009), this tool helps capture the state and development of a given research field.

The following SCOPUS search strategy was employed, incorporating commonly used terminology for homelessness and substance use:

(“substance use” OR “substance abuse” OR “psychoactive substances”) AND (“homeless individuals” OR “street dwellers” OR “street people”) AND “Colombia” AND (“factors influencing” OR “determinants” OR “causes”) AND (LIMIT-TO (DOCTYPE, “ar”)) AND (LIMIT-TO (LANGUAGE, “English”) OR LIMIT-TO (LANGUAGE, “Spanish”)) AND (LIMIT-TO (RCTYPE, “j”)) AND (LIMIT-TO (PUBSTAGE, “final”))

This search yielded 14 articles when limited to Colombia and 1,194 articles when conducted globally. Two tables were generated to summarize the retrieved documents, including authors, titles, abstracts, and keywords.

**Figure 1:**
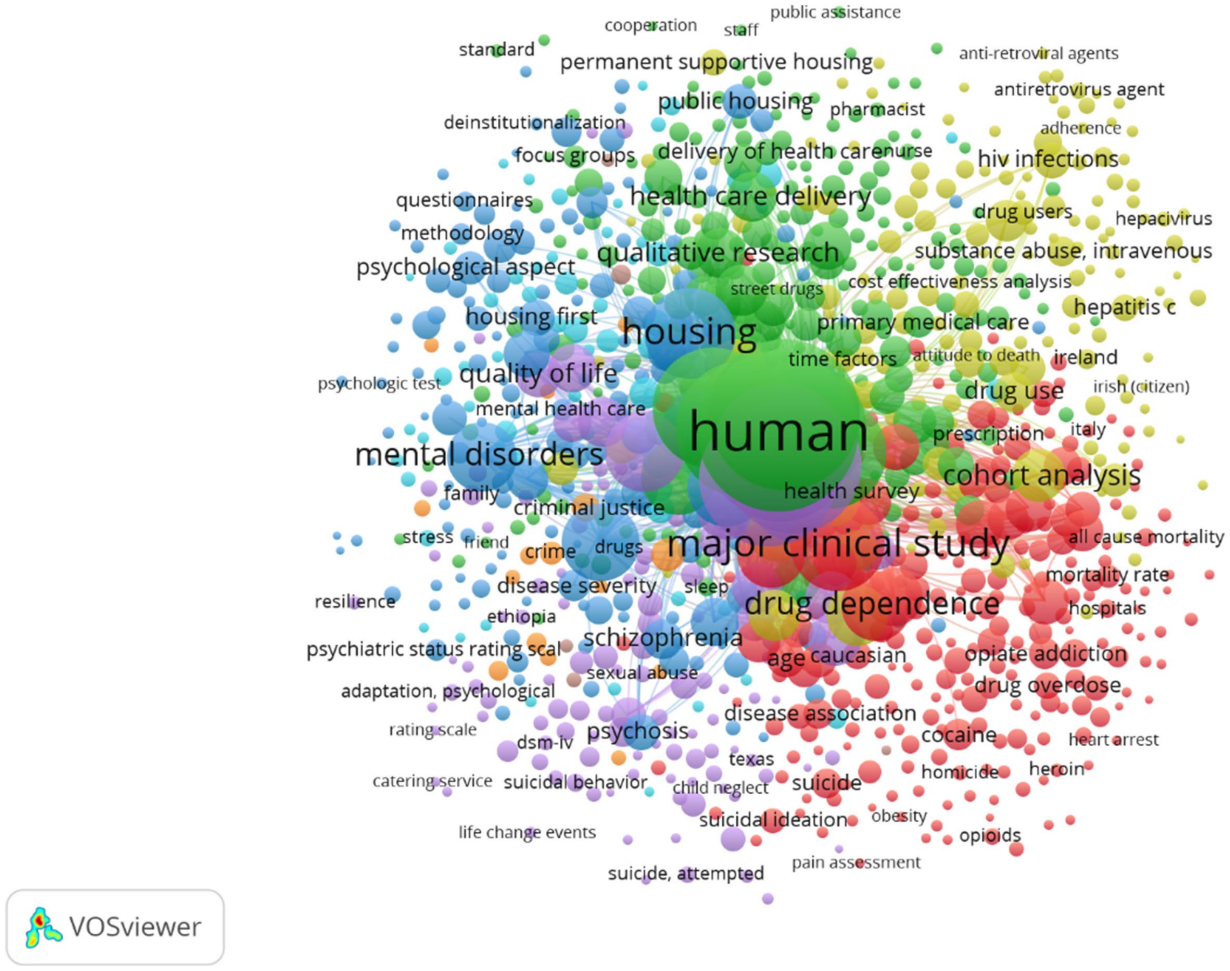
Keyword co-occurrence visualization network. Source: elaborated by the authors using VOSviewer.

Figure 1 presents a keyword co-occurrence network based on the 1,194 global articles. A threshold of at least two co-occurrences was set to ensure meaningful associations (Donthu et al., 2021). Eight distinct clusters emerged:

- Green cluster: focuses on improving access to stable housing and health services.
- Light blue cluster: examines the relationship between stable housing and mental health.
- Red cluster: emphasizes the public health implications of substance use.
- Yellow cluster: investigates drug-related diseases and transmission.
- Dark blue cluster: explores connections between mental health care and service provision.
- Lilac cluster: highlights the consequences of substance abuse, including mental disorders and reduced quality of life.
- Orange cluster: examines the impact of substance use on homeless populations.
- Brown cluster: focuses on the design and formulation of public policies.

These clusters illustrate the multidimensional nature of the relationship between homelessness and substance use. Several studies link trauma, early adversity, and housing instability with addiction and mental illness. Guillén et al. (2020) found that early-life stress increases the likelihood of substance use; Adams et al. (2023) emphasized substance use as a coping strategy; and Padgett (2020) framed these dynamics within structural public health failures. These findings support trauma-informed and housing-first interventions (Adams et al., 2023; Guillén et al., 2020; Padgett, 2020).

A second mapping exercise using the 14 Colombian articles showed that most studies were qualitative and focused on public health, homelessness, and the COVID-19 pandemic. This gap underscores the need for modeling approaches to better understand the multifactorial nature of substance use in this population.

**Figure 2:**
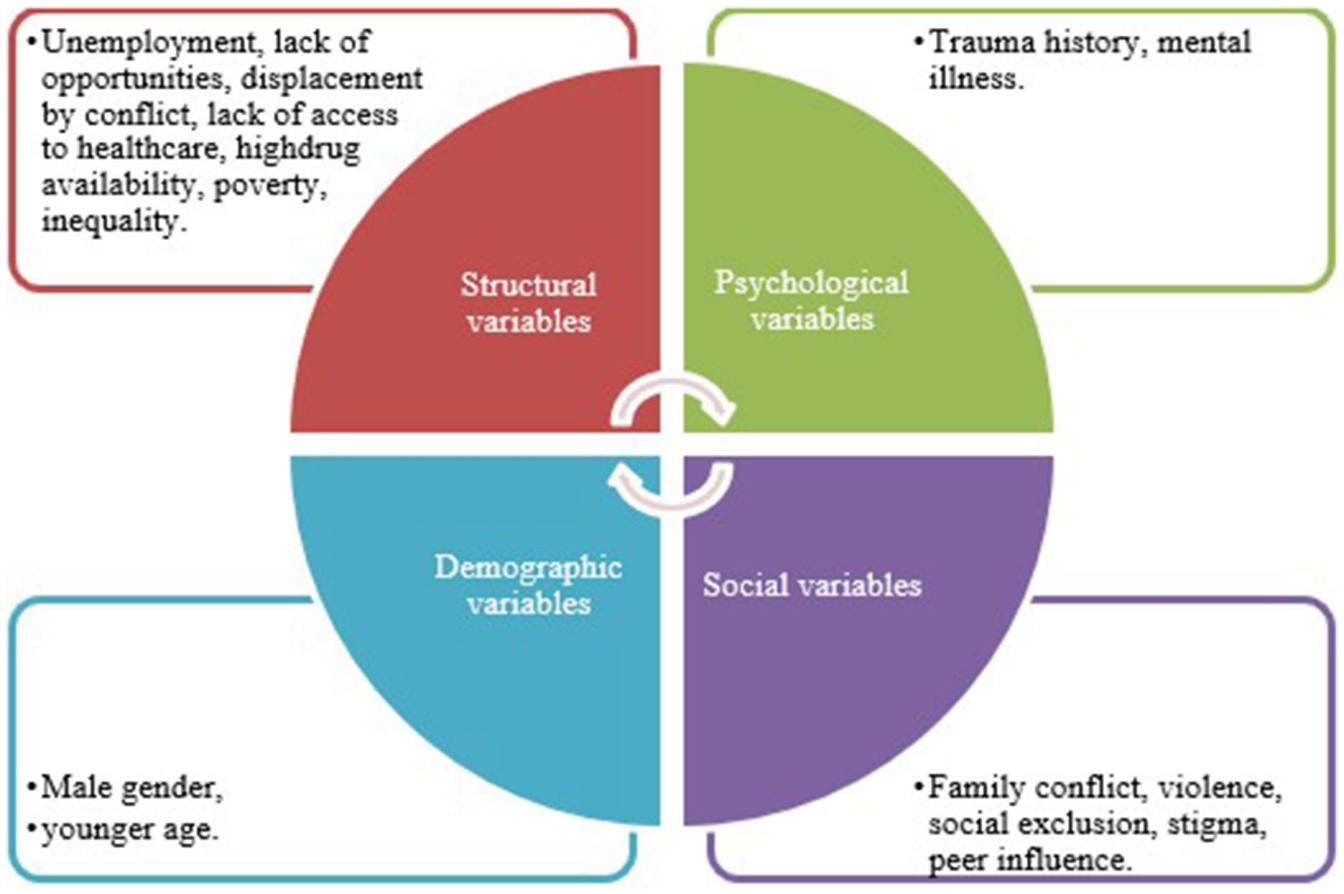
Conceptual model of initial variables influencing psychoactive substance use among people experiencing homelessness in Colombia. Source: elaborated by the authors.

Figure 2 presents a conceptual model based on the Colombian literature, highlighting how personal history, structural exclusion, and systemic inequality reinforce substance use trajectories. Adams et al. (2023) noted the enduring mental health impact of trauma experienced during homelessness. Aldridge (2020) argued that homelessness reflects broader social injustices, and that problematic substance use cannot be isolated from contexts of poverty and institutional neglect.

Integrating these insights through modeling allows for a nuanced understanding of homelessness and substance abuse—not merely as individual conditions, but as systemic phenomena embedded in cycles of exclusion. This understanding supports the development of more holistic and effective public health policies (Camargo et al., 2020; Fitzpatrick et al., 2013; Johnson and Chamberlain, 2008).

## 3 Methods

This study employed a quantitative methodology comprising two main stages: (1) a descriptive analysis of the homeless population in Colombia using data from the national censuses conducted by DANE (2017a, 2017b, 2019, 2021), and (2) the modeling of substance use predictors using the Random Forest algorithm.

### 3.1 Sample population

In accordance with Article 4 of Law 1,641 of 2013, DANE conducted three national censuses of individuals experiencing homelessness in 2017, 2019, and 2021 (DANE, 2017a, 2019, 2021). These were not based on probabilistic sampling but were full enumerations designed to collect demographic, social, and economic data in municipalities with significant homeless populations. The aim was to generate policy-relevant data to guide comprehensive public service planning for this population group.

The total number of individuals identified in each census was 9,538 in 2017, 13,252 in 2019, and 6,248 in 2021. Since Law 1,641 of 2013 mandates national-level policy formulation for homeless individuals, DANE is responsible for analyzing the demographic and socioeconomic characteristics of this group across all Colombian municipalities. The observed reduction in surveyed homeless individuals in 2021 reflects methodological differences in census coverage, with large urban centers - which typically concentrate homeless populations - being underrepresented relative to smaller municipalities in this wave. While the 2021 census expanded to include less populated Colombian municipalities compared to the 2017 and 2019 surveys, this geographical variation does not constitute a study limitation as our analysis revealed no significant spatial disparities in the outcomes.

The 2021 census was conducted during the COVID-19 pandemic, a period characterized by substantial disruptions to social services and population mobility patterns. While the official methodology documentation (DANE, 2021) does not explicitly describe pandemic-related adaptations to homeless enumeration protocols, the pandemic context likely influenced both the distribution and visibility of homeless populations - particularly through shifts from urban centers to peripheral areas (Ministerio de Salud y Protección Social, 2020). Therefore, year-to-year comparisons (2017–2021) should be interpreted with caution given these methodological and contextual considerations.

### 3.2 Data collection and variables

DANE collected census data through several methods: direct street-level outreach, fixed-point intercepts, mobile service brigades, and visits to institutions serving homeless populations. Structured questionnaires captured data in the following domains: (1) demographic and general information: Age, sex, literacy, education level, ethnicity, and place of birth; (2) substance use: Self-reported use of psychoactive substances; (3) contextual factors: Reasons for becoming or remaining homeless; (4) social ties: Receipt of aid, family interaction, and participation in municipal support programs; (5) safety and violence: exposure to threats or violence (DANE, 2017a, 2019, 2021). It is important to note that self-reported data on substance use may be subject to social desirability bias, potentially leading to underreporting, especially for substances considered more stigmatized or illicit.

### 3.3 Data processing and modeling

Data from the three censuses were analyzed in two stages: descriptive statistics and predictive modeling using decision trees and random forests.

#### 3.3.1 Descriptive analysis

The descriptive analysis focused on key variables: age, sex, education level, literacy, reason for entering homelessness, duration of homelessness, reason for remaining homeless, family contact, participation in support programs, and income generation. Particular attention was given to the nine psychoactive substances identified by DANE: Cigarettes, alcohol (including antiseptic alcohol mixed with beverages), marijuana, inhalants (e.g., sacol, glue, thinner), cocaine, basuco (impure cocaine sulfate), heroin, pills, and other substances (e.g., *maduro*, *pistolo*). These variables were analyzed both individually and in combination to explore potential correlations and patterns.

While acknowledging the relevance of variables such as mental health status, history of trauma, and social support networks (as well as forms of homelessness not typically captured through street-based enumeration, such as hidden homelessness or temporary accommodations, which the literature recognizes as important predictors) this study intentionally focuses on the variables available in the DANE censuses (DANE, 2017a, 2019, 2021). This methodological choice ensures consistency and replicability based on the most comprehensive national data currently available, while also recognizing that future research could enrich these findings by incorporating additional psychosocial and contextual dimensions through complementary approaches.

While the DANE censuses (DANE, 2017a, 2019, 2021) provide valuable data for public policy, its methodological documentation does not explicitly reference ethical procedures such as informed consent, confidentiality safeguards, or protection protocols tailored to vulnerable populations. Although DANE has general guidelines on microdata anonymization (DANE, 2017b), their absence from specific documentation highlights the opportunity to strengthen future statistical operations with explicit ethical frameworks that are sensitive to the structural conditions of the surveyed population and aligned with international standards.

#### 3.3.2 Predictive modeling

For the predictive analysis, the decision trees and random forests were implemented to identify the most significant predictors of psychoactive substance use among homeless individuals. This approach draws on prior studies using machine learning to detect early indicators of homelessness (Yoder et al., 2021).

The modeling utilized demographic data and substance use responses from the 2017, 2019, and 2021 censuses. The Random Forest algorithm was employed to distinguish between users and non-users, based on the demographic and social variables outlined above.

Decision Trees were selected for their interpretability and ability to define subgroups within the homeless population. The Gini index (Atkinson and Bourguignon, 2012) was used as the criterion for node splitting, measuring impurity. Nodes with homogeneous substance use patterns (e.g., all users or all non-users) had a Gini index close to 0, whereas heterogeneous nodes approached a value of 1. This measure guided the selection of optimal splits, identifying the most informative demographic variables.

The algorithm began with a root node reflecting the initial distribution of substance users and non-users, then applied the Gini-based split rule iteratively to generate branches. The process continued until one of the stopping conditions was met—either reaching maximum tree depth or achieving a Gini index of 0.

Random Forests extended this approach by training multiple decision trees using randomly selected subsets of both the dataset and the variables. For each split in a tree, a subset of variables was considered, and for each tree, a bootstrap sample of the dataset was drawn. Predictions were aggregated using majority voting.

**Figure 3:**
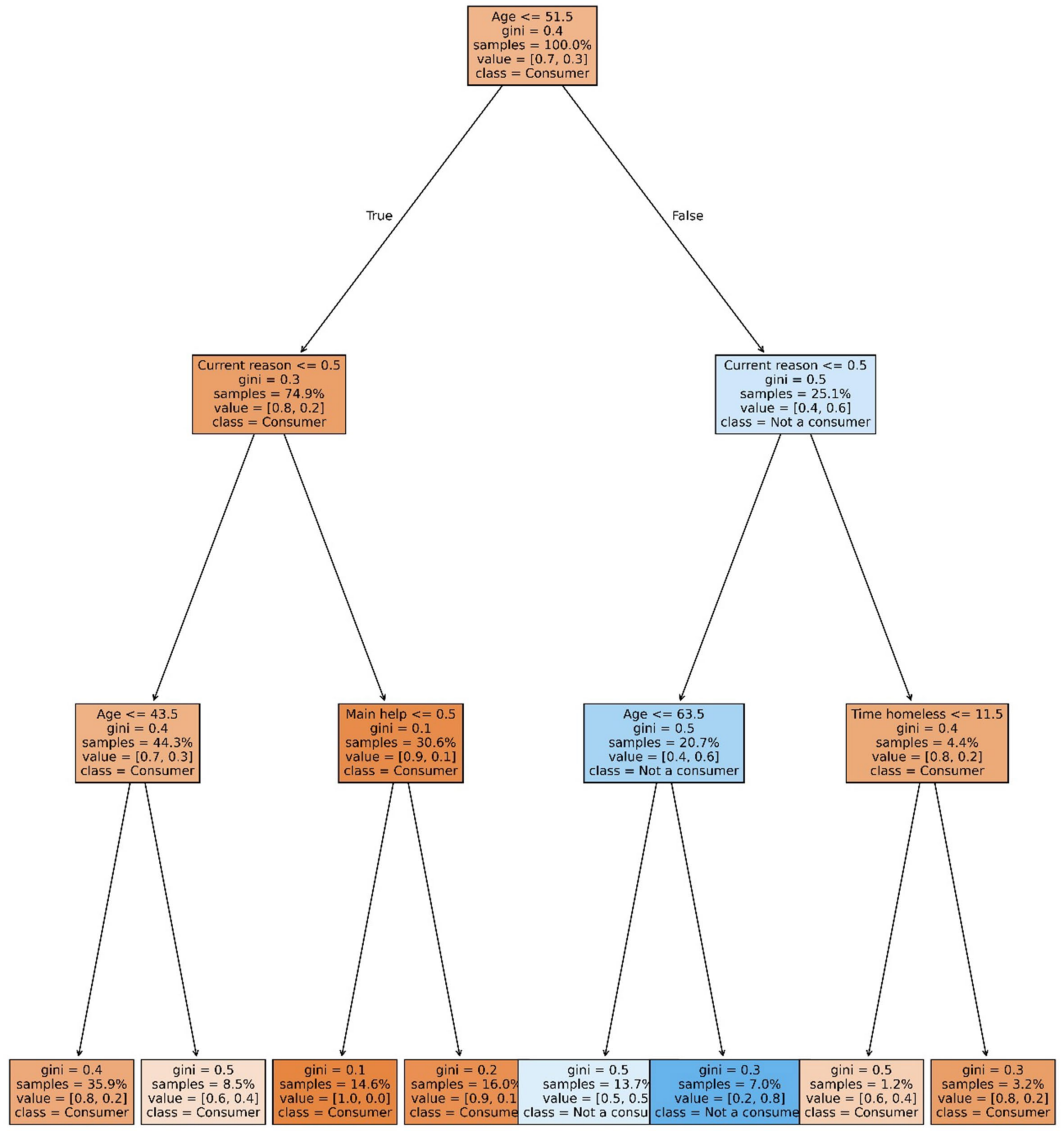
General consumption decision tree. Source: elaborated by the authors using the Python Sklearn database.

**Figure 4:**
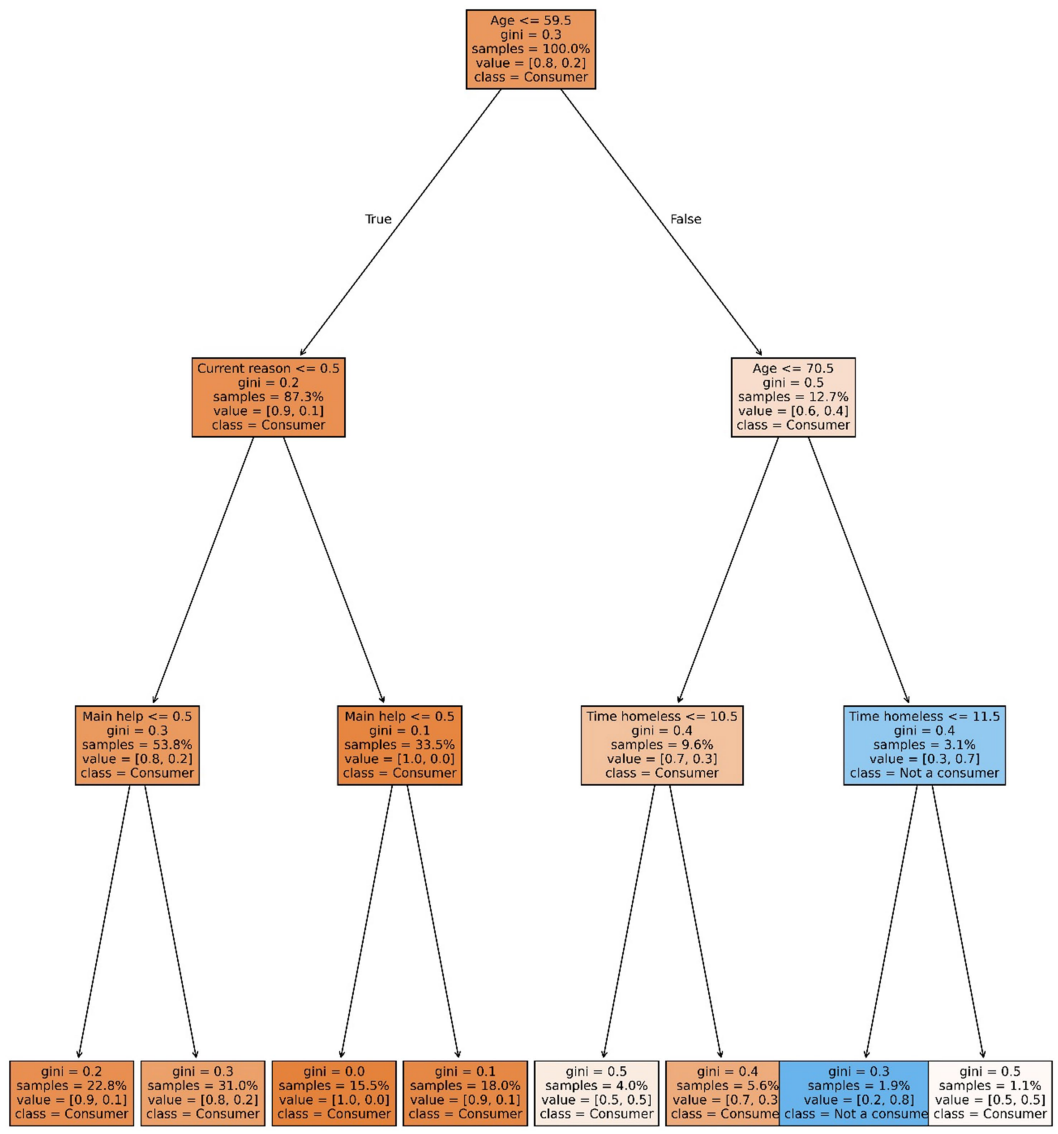
Heavy consumption decision tree. Source: elaborated by the authors using the Python Sklearn library.

The implementation used the *DecisionTreeClassifier* from the *sklearn.tree* library. Trees were configured with a maximum depth of 3 and a minimum of five observations per leaf. To avoid overfitting and enhance generalizability, the Random Forest ensemble was tuned with a maximum depth of 20 and the same minimum leaf size. Hyperparameter tuning was performed across a range of depths (5–20) (Figures 3, 4).

The data was partitioned into training (80%) and testing (20%) subsets. The Gini index was retained as the splitting criterion. Learning curves were generated to assess model behavior and detect signs of overfitting or under fitting.

This modeling framework allowed for the identification of key predictors of substance use. These include education level, reason for becoming homeless, duration of homelessness, age, sex, family contact, literacy, support program participation, and income source. The models were configured to provide actionable insights into how these factors relate to both general and heavy substance use, thereby informing public policy design as outlined in Law 1641 of 2013.

Finally, while this study relied on Random Forest for predictive purposes, future studies may benefit from integrating spatial methods such as Geographically Weighted Regression (Roy et al., 2024a), which allow for geographic variation in vulnerability and substance use behavior.

### 3.4 Interactive dashboard

An interactive dashboard was developed in Tableau Public to disseminate the findings. The dashboard displays disaggregated information by year, municipality, education level, type of substance consumed, reasons for homelessness, sex, age group, and duration of homelessness (González, 2024). This tool serves as a resource for researchers, policymakers, and service providers addressing homelessness in Colombia.

## 4 Results

This section presents the results of the descriptive analysis and predictive modeling of psychoactive substance use among individuals experiencing homelessness in Colombia, based on census data from 2017, 2019, and 2021.

### 4.1 Substance use classification

The DANE census examined nine types of psychoactive substances. To analyze consumption patterns, two analytical categories were created:

1. General users of psychoactive substances (Group 1): individuals who reported using any of the nine substances, including cigarettes and alcohol.
2. Heavy users of psychoactive substances (Group 2): individuals who reported using at least one of the following: marijuana, inhalants, cocaine, basuco, heroin, pills, or other substances. This group excludes those who reported using only cigarettes or alcohol.

This classification distinguishes between socially accepted substances (such as alcohol and tobacco) and those more strongly associated with social stigma and health deterioration, including inhalants, cocaine, and heroin. This distinction is supported by studies such as Ballard et al. (2023), who document how the use of high-risk injectable substances among people experiencing homelessness in rural areas of the United States is linked to structural barriers in accessing health services, as well as increased exposure to drug-related harms. In this sense, their findings support the notion that certain patterns of substance use (beyond their legal status) are closely tied to processes of social exclusion and structural vulnerability. Thus, while alcohol or cigarette use in contexts of homelessness does not necessarily imply problematic consumption, the use of high-risk illicit drugs clearly indicates greater social and health-related risks. This aligns with literature that differentiates between socially tolerated forms of consumption and those characteristic of marginalized groups (Ballard et al., 2023; Coombs et al., 2024).

Across the three censuses, the proportion of individuals who reported using at least one substance (Group 1) ranged from 66 to 68%. When excluding alcohol and cigarette users (Group 2), the proportion ranged from 56 to 58%.

### 4.2 Demographic patterns

Men consistently represented most individuals experiencing homelessness (88–89%), as well as most general and heavy substance users (88–90%). Approximately 34% of individuals reported living in a municipality different from their place of birth, 43% remained in their place of birth, and 23% had no recorded relocation information. A total of 264 minors were identified, indicating that about 1% of the homeless population is underage.

The main factors cited for entering homelessness were: Psychoactive substance use (36.6%); Family conflict or difficulties (30.1%); Economic hardship (9.2%).

The main reasons for remaining homeless were: Continued substance use (36.7%); Economic hardship (14.1%); Family conflict or difficulties (12.3%); Personal choice (12.2%).

Among adults, 35% reported becoming and remaining homeless due to substance use. Among youth, this percentage was higher: 41% became homeless due to substance use, and 43% remained so for the same reason, indicating a heightened vulnerability in younger populations.

### 4.3 Predictive modeling results

The modeling was performed on a filtered sample of 11,925 individuals who had complete data for key demographic, behavioral, and contextual variables. These included age, sex, education level, literacy, place of origin, housing location, reasons for becoming and remaining homeless, duration of homelessness, family contact, receipt of aid, income generation, and sexual orientation.

Decision tree results:

- General users: the model predicted substance use in 7 out of 8 leaf nodes. Only node 7—representing individuals over age 70 who had experienced homelessness for 10 years or less—predicted non-use, comprising 1.9% of the sample with a predicted probability of 0.8 of being non-users.
- Key finding: substance use was pervasive across nearly all demographic groups, regardless of sex, education level, age, or access to family or state support.
- Nodes 1–4 (87.3% of the sample) predicted high probability of general use among individuals under 60 who did not specify reasons for homelessness.
- Heavy users: node 6 predicted non-heavy use with a probability of 0.8 among individuals aged 64 or older who did not cite substance use as a reason for becoming or remaining homeless. Node 5 (younger individuals with similar profiles) showed high uncertainty. Node 4 predicted a high probability of heavy use among individuals under 50 who cited continued consumption and reported no institutional aid.

**Figure 5:**
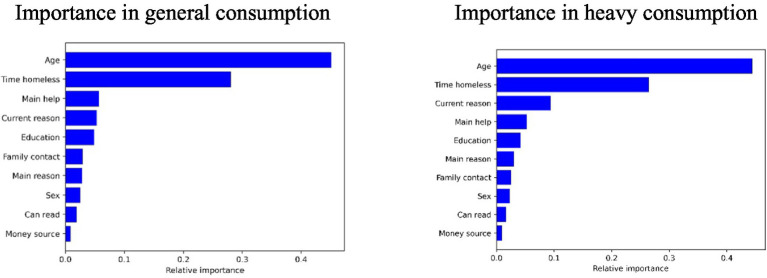
Importance of the variables in the consumption of psychoactive substances. Source: elaborated by the authors using the Python Sklearn library.

Variable importance (Figure 5):

- Age: the most influential predictor for both general and heavy use. Younger individuals had higher consumption rates.
- Duration of homelessness: second most influential predictor. Shorter durations were associated with lower consumption.
- Other influential variables (in decreasing order of importance): sex, education level, literacy, reason for homelessness, family interaction, participation in support programs, and income source.

Demographic trends:

- Average age of general users: 39.2 years
- Average age of non-users: 49.4 years
- Women were slightly younger on average than men.
- Women who used substances were younger than women who did not.

This suggests a potential age-related decline in substance use. However, this trend may be influenced by survivorship bias—older individuals experiencing homelessness may have higher mortality, which reduces their representation in the data.

### 4.4 Model performance

**Figure 6:**
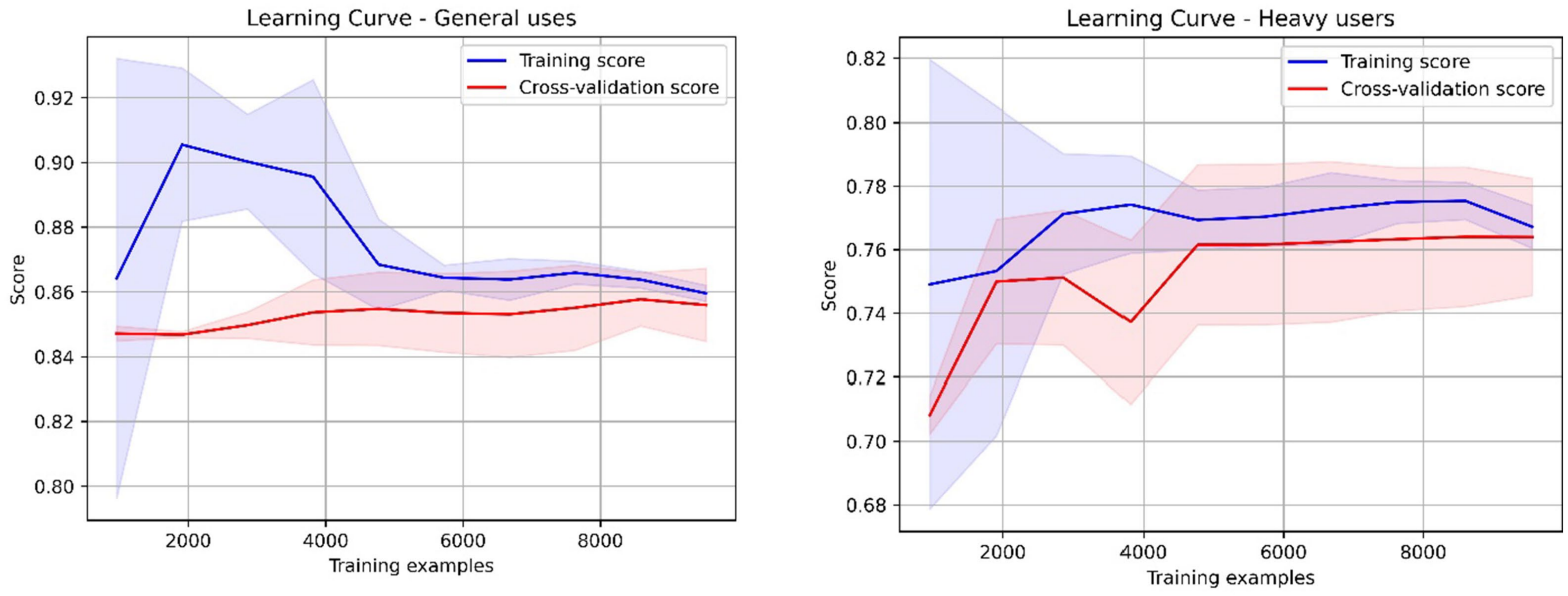
Learning curves. Source: elaborated by the authors using the Python Sklearn library.

Figure 6 presents the learning curves for the predictive models trained on two distinct cohorts: general users and heavy psychoactive substance users. Both curves exhibit convergence between training and validation performance metrics, although this convergence is more pronounced in the general user model. For this model, training accuracy stabilizes at approximately 86% (SD ± 0.3%), while validation accuracy reaches 85.6% (SD ± 0.7%), indicating a narrow generalization gap when the full dataset is utilized. This suggests that the model effectively captures generalizable patterns within the population, with diminishing gains observed as additional training data are introduced.

In contrast, the heavy substance user model shows a slightly wider generalization gap—training accuracy of 76.6% (SD ± 0.5%) and validation accuracy of 76.3% (SD ± 1.8%)—which may reflect greater inherent variability in the behavioral patterns of this subgroup, or limitations related to sample size.

The general use model achieved an overall accuracy of 85.8% based on 2,385 observations. However, performance varied substantially between classes. The majority class (Consumer, 2,240 cases) exhibited high precision (0.97), recall (0.88), and F1-score (0.92), while the minority class (Not a consumer, 145 cases) showed low precision (0.23) despite moderate recall (0.57), resulting in a substantially lower F1-score (0.33). The macro-averaged F1-score (0.62) highlights challenges in generalizing across classes, while the weighted F1-score (0.88) reflects the predominance of the consumer class. The limited performance observed for the minority class (non-users) reflects a study limitation inherent to class imbalance within the dataset—an issue consistent with existing epidemiological evidence. Large-scale studies have shown that complete abstinence from psychoactive substances is uncommon among homeless populations. For instance, Henwood et al. (2023), in a multicenter study of 2,873 homeless adults, reported that only 9.2% had not used any substances in the preceding year. This demographic reality inherently constrains the predictive performance for non-users, as the class distribution in our dataset closely reflects real-world prevalence patterns.

The heavy use model yielded an overall accuracy of 76.3% (2,385 cases). For the consumer class (1,870 cases), the model demonstrated high precision (0.89) and moderate recall (0.80), resulting in an F1-score of 0.84. The minority class (Not a consumer, 515 cases) exhibited lower precision (0.46) but higher recall (0.64), producing an F1-score of 0.54. The macro-averaged F1-score (0.69) indicates moderate generalization across classes, while the weighted F1-score (0.78) better reflects the model’s performance within the more prevalent consumer class.

**Figure 7:**
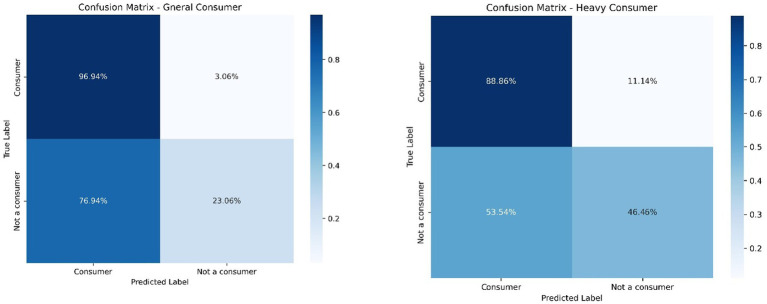
Confusion matrices. Source: elaborated by the authors.

Figure 7 displays the confusion matrices for both models, illustrating their classification performance by actual versus predicted class labels.

### 4.5 Cross-tabulation insights

Table 3 cross-references initial and sustained reasons for homelessness. Notably, those who reported becoming homeless due to substance use overwhelmingly remained so for the same reason. Among individuals who became homeless due to family conflict, 39% cited the same issue for remaining homeless, but a significant share (30%) reported continued substance use as the sustaining factor. Economic hardship, though a frequent cause of initial homelessness, was less often linked to ongoing substance use (Table 4).

**Table 3:** Consumption of psychoactive substances according to age.

Consumer	Answer	Average age
General	Yes	39,2
	No	49,4
Heavy	Yes	37,6
	No	48,9

Source: elaborated by the authors.

**Table 4:** Relationship between the reason why people began to experience homelessness and the reason why they continued in that situation.

Main reason why people began to experience homelessness	Main reason why people continue to experience homelessness			
	Family conflicts or difficulties	Consumption of psychoactive substances	Economic difficulties	Personal choice
Family conflicts or difficulties	39%	30%	16%	16%
Consumption of psychoactive substances	6%	81%	6%	8%
Economic difficulties	4%	8%	83%	4%

Source: elaborated by the authors.

These findings underscore the cyclical and compounding relationship between substance use and homelessness.

## 5 Discussion

This study sought to answer the question: What are the variables that affect the consumption of psychoactive substances among individuals experiencing homelessness in Colombia? The findings revealed that age and the duration of homelessness are the two most influential factors in predicting both general and heavy substance use. Additionally, sex emerged as a relevant factor: men are more likely to use substances, consistent with the findings of Coombs et al. (2024) and Laporte et al. (2018), who identified alcohol as the most consumed substance among this population (Table 5).

**Table 5:** Metrics random forest.

General consumer	Precision	Recall	f1-score	Support
Consumer	0.969383 ± 0.02	0.87634	0.92052	2,240
Not a consumer	0.230556 ± 0.09	0.57241	0.32871	145
accuracy	0.857862	0.85786	0.85786	0.85786
macro avg	0.599969	0.72438	0.62461	2,385
weighted avg	0.924465	0.85786	0.88454	2,385

Heavy consumer	Precision	Recall	f1-score	Support
Consumer	0.888624 ± 0.02	0.79786	0.8408	1870
Not a consumer	0.464589 ± 0.04	0.63689	0.53727	515
accuracy	0.763103	0.7631	0.7631	0.7631
macro avg	0.676607	0.71738	0.68903	2,385
weighted avg	0.797061	0.7631	0.77526	2,385

Source: elaborated by the authors.

The importance of incorporating a gender perspective into both research and policy is increasingly recognized. Scholars such as Galán-Sanantonio et al. (2024), Laporte et al. (2018), Roy et al. (2022), and Guillén et al. (2020) stress the need to address the differentiated pathways through which men and women experience homelessness. While men are more likely to live on the streets, women more often experience hidden homelessness (e.g., staying with acquaintances or in temporary housing). This distinction underscores the necessity of gender-sensitive public policies that account for varied experiences and risks.

Age also emerged as a significant predictor. Fitzpatrick et al. (2013) found that adverse childhood conditions are strongly associated with multiple exclusion events and homelessness later in life. In our study, younger individuals facing economic instability, family conflict, or mental health challenges were more vulnerable to both homelessness and substance use. Older individuals, in contrast, often confront chronic health conditions and barriers to reentering stable housing, a finding that aligns with Aldridge et al. (2018) on the compounded health risks of prolonged homelessness.

These findings also correlate with the thematic clusters identified in the bibliometric mapping (Figure 1), particularly those emphasizing the intersection between age and mental health (light blue cluster), the harmful effects of substance use (orange cluster), and the role of public policy (brown cluster).

A major strength of this study is its reliance on nationwide census data provided by DANE, which offers a broad and representative snapshot of the homeless population in Colombia. Furthermore, the application of machine learning techniques—including decision trees and random forests—enabled the identification of variables with the strongest predictive power for substance use.

However, this study is not without limitations. First, it is exploratory in nature and does not aim to test formal hypotheses. Second, while the results provide robust predictive insights, they do not uncover the causal mechanisms that underpin the observed relationships. Third, qualitative research would be valuable to deepen the understanding of how structural, psychosocial, and policy-level factors interact in shaping the lived experiences of substance use and homelessness.

It is important to clarify that, although the statistical models identify strong associations between certain variables and substance use, these findings should not be interpreted as evidence of causal relationships. The cross-sectional nature of the data limits the ability to establish temporal or causal mechanisms. This study also underscores the importance of analyzing the bidirectional relationship between homelessness and substance use. As suggested by Johnson and Chamberlain (2008), these factors reinforce one another in a feedback loop: substance use can lead to homelessness, while the harsh conditions of homelessness can exacerbate substance dependence. Future research could build on this dynamic by incorporating system dynamics modeling (e.g., Bala et al., 2017; Aracil and Gordillo, 1997), as in the study by Jaén and Dyner (2007), who used this methodology to simulate drug market behavior over time. The findings of this study have limited generalizability beyond the 78 Colombian municipalities included in the homeless censuses 2017, 2019 and 2021. While the identified associations between variables provide important insights into local patterns, they may not be representative of other municipalities in Colombia or different regions of Latin America.

While the present study primarily focuses on demographic and behavioral variables, future work should also consider spatial inequalities. Roy et al. (2024b), for example, employed multivariate geographically weighted regression (MGWR) to examine regional disparities in wealth, which could also inform studies on the territorial dimension of homelessness and substance use in Colombia.

Finally, the findings offer practical implications for public policy and intervention strategies. The strong association between younger age, short duration of homelessness, and substance use highlights the importance of early intervention. Preventive efforts (including educational programs, vocational training, and targeted substance abuse prevention) should focus on individuals in the early stages of homelessness. Such efforts could reduce the risk of long-term exclusion and facilitate reintegration.

Given the legal mandate under Colombian law to provide comprehensive care for people experiencing homelessness, we recommend further evaluation of models such as those proposed by Yoder et al. (2021), which use predictive modeling to inform service delivery. Coordinated efforts among government agencies, academic institutions, and civil society are essential to implementing effective, evidence-based strategies. Moreover, sustained investment in prevention is key to reducing the likelihood that individuals will enter or remain in homelessness due to substance use.

## 6 Conclusion

This study presents an innovative application of data science and machine learning techniques to model the variables associated with psychoactive substance use among individuals experiencing homelessness in Colombia. By using census data collected by DANE in 2017, 2019, and 2021, this research provides a detailed, data-driven analysis of the demographic and behavioral factors strongly associated with substance use in this vulnerable population.

The findings demonstrate that age is a key differentiating variable. The average age of individuals who do not consume psychoactive substances is significantly higher (49.4 years) than that of general users (39.2 years). This age-related difference remains consistent even when comparing heavy users with non-users. The analysis also revealed that women experiencing homelessness tend to be slightly younger than men, and that women who use substances are significantly younger than those who do not.

A notable pattern that emerged from the results is the potential decline in substance use with age, possibly due to survival bias, as individuals who have consumed heavily over time may face higher mortality risks and thus be underrepresented in later age groups.

In addition to age, the duration of homelessness also emerged as one of the most important predictors of substance use. Shorter durations were associated with lower rates of both general and heavy substance use. This suggests that interventions targeting individuals early in their homelessness trajectory may be more effective at preventing or reducing substance dependence.

These findings have important implications for public policy design. Modeling the variables that influence substance use allows for more precise, evidence-based interventions to support reintegration, improve access to services, and protect the rights of individuals experiencing homelessness. The early identification of at-risk individuals could help optimize resource allocation and maximize the impact of prevention and rehabilitation programs.

In alignment with Law 1641 of 2013, which mandates the comprehensive care and inclusion of people experiencing homelessness in Colombia, the study supports the development of targeted strategies that consider the demographic, behavioral, and structural dimensions of homelessness and substance use. Future research could expand this work by incorporating dynamic modeling techniques and spatial analyses to further understand how substance use behaviors evolve over time and across regions.

## Data Availability

The original contributions presented in the study are included in the article/supplementary material, further inquiries can be directed to the corresponding author.
